# Contralateral Primary Small-Cell Neuroendocrine Carcinoma of the Breast With Focal Squamous Differentiation in a Patient With Prior Invasive Ductal Carcinoma: A Rare Case

**DOI:** 10.7759/cureus.115114

**Published:** 2026-08-24

**Authors:** Erik L Parkhurst, Amar Hamad

**Affiliations:** 1 International Program, Universidad Autónoma de Guadalajara (UAG) School of Medicine, Zapopan, MEX; 2 Hematology and Medical Oncology, Affiliated Oncologists, Christ Medical Center, University of Illinois Chicago, Oak Lawn, USA

**Keywords:** breast neuroendocrine carcinoma, breast pathology, case report, contralateral breast cancer, neuroendocrine differentiation

## Abstract

Primary neuroendocrine carcinoma of the breast is a rare malignancy. Tumors demonstrating predominant neuroendocrine differentiation with focal squamous features are exceptionally uncommon, and metachronous contralateral development following previously treated invasive ductal carcinoma (IDC) has been reported only rarely. We present the case of a 68-year-old woman with a history of estrogen receptor-positive, HER2-negative left breast IDC treated with neoadjuvant chemotherapy, mastectomy, adjuvant radiation therapy, and endocrine therapy who developed a distinct contralateral high-grade breast carcinoma seven years later. Following the discovery of right axillary lymphadenopathy, imaging revealed a right breast mass with progressive axillary adenopathy. Histopathologic examination demonstrated a primary small-cell neuroendocrine carcinoma of the breast with focal squamous differentiation. Immunohistochemistry showed diffuse positivity for INSM1, synaptophysin, and p63, with negativity for estrogen receptor, progesterone receptor, HER2, TTF-1, CDX2, SOX10, and GATA3, supporting a primary breast origin. This case highlights the diagnostic complexity and therapeutic uncertainty surrounding a metachronous contralateral neuroendocrine breast carcinoma following treatment of a separate primary breast malignancy. This exceptionally rare entity underscores the importance of continued case reporting to improve understanding of its clinicopathologic features and guide future management strategies.

## Introduction

Primary small-cell neuroendocrine carcinoma of the breast with focal squamous differentiation represents an exceptionally uncommon presentation. The 2026 World Health Organization classification of breast tumors revised the terminology and diagnostic approach for breast tumors exhibiting neuroendocrine morphology, emphasizing the distinction among primary small-cell neuroendocrine carcinoma, invasive breast carcinoma no special type with neuroendocrine differentiation, primary breast neuroendocrine tumor, and metastatic neuroendocrine carcinoma involving the breast [1-4]. In the present case, the tumor demonstrated small-cell morphology with predominant neuroendocrine differentiation and focal squamous differentiation. Diffuse INSM1 and synaptophysin expression supported neuroendocrine differentiation, while p63 and focal CK5/6 expression supported squamous differentiation. The absence of TTF-1, CDX-2, GATA-3, and SOX10 expression, together with clinical and imaging correlation, favored a primary breast origin. The tumor was therefore considered most consistent with a primary small-cell neuroendocrine carcinoma of the breast with focal squamous differentiation.

Invasive ductal carcinoma (IDC) is the most prevalent histologic subtype of breast carcinoma, accounting for an estimated 80% of all invasive breast cancers [5,6]. Invasive breast cancers are characterized by neoplastic cells invading beyond the basement membrane and exhibit morphological variability [7]. Subtypes are identified using a range of testing modalities, including staining for hormone receptors, HER2 receptors, tumor grade, pleomorphism, multifocality, morphology, and Ki-67 index.

Contralateral breast cancer is a second primary breast cancer occurring in the opposite breast of a patient with previously diagnosed or treated unilateral breast cancer. The incidence of this type of breast cancer has declined in recent years due to treatment advancements, particularly with systemic therapies. Still, its occurrence remains important in discussions of survival outcomes and quality of life [8]. Typically, contralateral breast cancers are considered second primary malignancies distinct from the initial tumor; however, in some cases, these cancers can share clonal similarities with the original tumor as a possible manifestation of micrometastatic disease [9,10]. Histologically distinct contralateral tumors are, however, exceedingly uncommon, particularly those exhibiting divergent differentiation [11-14].

Here, we describe a rare case of a 68-year-old woman with a prior history of treated IDC of the left breast who developed a contralateral primary small-cell neuroendocrine carcinoma of the breast with focal squamous differentiation. We discuss the clinicopathologic features, diagnostic patterns, and treatment options to consider when managing this unusual presentation.

## Case presentation

Our case concerns a 68-year-old woman with a past medical history of type 2 diabetes mellitus with peripheral neuropathy, hypertension, obesity, hypothyroidism, asthma, and stasis dermatitis with venous insufficiency, who was originally referred to the multidisciplinary oncology clinic in the summer by her primary care provider with new-onset complaints of nipple inversion and bloody discharge from the left nipple with an abnormal mammogram. She had a remote history of an additional abnormal mammogram with a benign right breast biopsy 12 years earlier and underwent annual mammography until the new left breast abnormality was identified. That mammogram revealed calcifications in the left upper outer quadrant with focal asymmetry at 3 o’clock and a 2.5-cm tumor, with axillary lymph node enlargement.

Other notable history included a patient-reported obstetric history of G0P0A0, menarche in her 50s with no history of systemic estrogen-only hormone replacement therapy, non-smoker, no history of drug use, and no other personal oncologic history. Family history was notable for a maternal aunt with breast cancer at the age of 80 and a maternal uncle with prostate cancer in his 60s. Given the family history, the patient underwent genetic testing, which was negative for any alterations in the following genes: APC, ATM, BARD1, BRCA1, BRCA2, BRIP1, CDH1, CDK4, CDKN2A, CHEK2, DICER1, HOXB13, MLH1, MRE11A, MSH2, MSH6, MUTYH, NBN, NF1, PALB2, PMS2, POLD1, POLE, PTEN, RAD50, RAD51C, RAD51D, SMAD4, SMARCA4, STK11, TP53, EPCAM, and GREM1.

An initial US-guided biopsy of the left breast demonstrated infiltrating ductal carcinoma grade 1, which was estrogen receptor positive at 100%, progesterone receptor positive at 30%, and HER2 negative with a Ki-67 of 10%. Multiple axillary lymph nodes were positive as well. Subsequent MRI also demonstrated multiple lesions in the left breast. PET-CT around the same time was otherwise unremarkable and showed no evidence of additional dissemination or distant metastasis.

With the patient’s estrogen receptor-positive tumor status, she was started on endocrine hormone-based therapy with anastrozole after the initial biopsy before the start of neoadjuvant chemotherapy. The goal of this therapy was to downstage the tumor and reduce its size to expand treatment options. She then had a vascular access device placed later that year, and neoadjuvant chemotherapy with docetaxel/cyclophosphamide (TC regimen) was initiated, with an expected duration of six cycles. On the recommendation of the multidisciplinary oncology team, after completing neoadjuvant chemotherapy, the patient underwent a left mastectomy early the following year. Pathological analysis from the mastectomy noted two distinct lesions; the first demonstrated infiltrating ductal carcinoma, grade 2, measuring 3 x 2 x 2 cm in greatest dimensions. The second lesion had a minor component of ductal carcinoma in situ, grade 2, solid type with focal necrosis, in and adjacent to the infiltrating component. The patient's mastectomy scar was determined to be well-healed by the subsequent spring.

Post-mastectomy, she underwent radiation therapy to the left chest wall. She underwent radiation therapy to the left chest wall and supraclavicular region from spring to summer of year 2, receiving a total of 35 treatments to each area, secondary to previously noted regional lymph node involvement, to destroy any potential microscopic tumor cells to improve regional control and overall survival rates. Additionally, she received four left mastectomy scar boost treatments in the summer of year 2 and utilized Domeboro soaks and Silvadene topical cream to promote healing.

The patient continued regular follow-ups as directed and restarted anastrozole in the summer of year 2. She had a right digital diagnostic mammogram later that fall, which showed no evidence of malignancy and recommended a repeat in one year. The patient remained well throughout follow-up and had no significant complaints.

She had a repeat right breast ultrasound in year 3, which again showed no sonographic evidence of malignancy. Another routine repeat right breast ultrasound in year 4 of care demonstrated scattered fibroglandular elements in the right breast, with a repeat requested approximately six months later. A subsequent right diagnostic mammogram at regular follow-up without new symptoms was then noted to show a cluster of coarse lucent-centered punctate calcifications in the right breast that resembled fibrocystic change or sclerosing adenosis. About six months later, a serial mammogram again showed a stable cluster of coarse lucent-centered punctate calcifications in the right breast at 12 o'clock posterior depth.

Given persistent changes from normal breast ultrasounds in years 2 and 3 and overall history, as a precaution, she underwent a mammogram with a stereotactic breast biopsy in year 5. Pathology results noted benign breast tissue with fibroadenomatous change and stromal fibrosis with prominent calcifications present, which was concordant with imaging findings. Another right breast mammogram in year 7 reported heterogeneously or extremely dense breast tissue without mammographic evidence of malignancy.

The patient was doing well without significant complaints or concerns throughout monitoring recommendations until she was seen in the late summer of year 7 with complaints of axillary lymph node enlargement on self-examination. She denied any additional symptoms such as nipple inversion, skin changes, or nipple discharge. A right breast mammogram with targeted ultrasound imaging of the right axilla demonstrated newly enlarged, abnormal right axillary lymph nodes with effaced fatty hila. A representative right axillary lymph node measured 2 x 1.5 x 1.3 cm in the right axilla, 18 cm from the nipple. The pathology report from the follow-up ultrasound-guided right axilla biopsy stated metastatic high-grade neuroendocrine carcinoma with necrosis, favoring small-cell type, within lymph node tissue. The tumor cells were positive for cytokeratin CAM5.2 and INSM1, weakly positive for CK7, synaptophysin, and PAX8, and negative for TTF-1, GATA-3, CDX-2, and SOX10. The tumor cells were negative for estrogen receptor (0%), progesterone receptor (0%), and HER2/neu (IHC score 0) and had a high proliferative index (Ki-67 80%). Per the report, the tumor cells showed morphologic features different from those observed in the prior contralateral breast resection.

**Figure 1:**
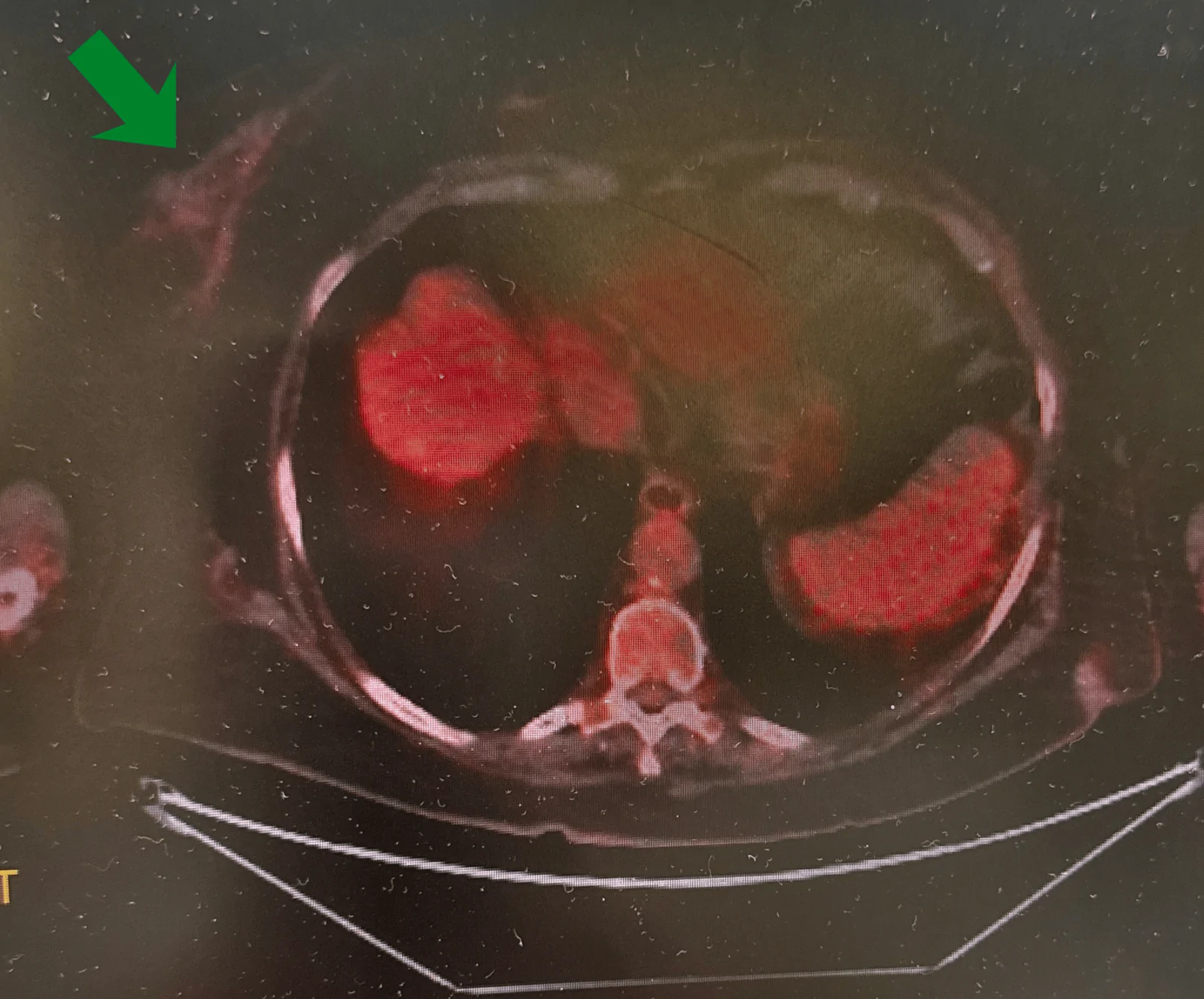
18F-FDG PET/CT demonstrating FDG-avid right breast malignancy Axial fused PET/CT image demonstrates an area of increased FDG uptake corresponding to the newly identified right breast lesion (green arrow). 18F-FDG: fluorine-18 fluorodeoxyglucose, PET/CT: positron emission tomography/computed tomography

**Figure 2:**
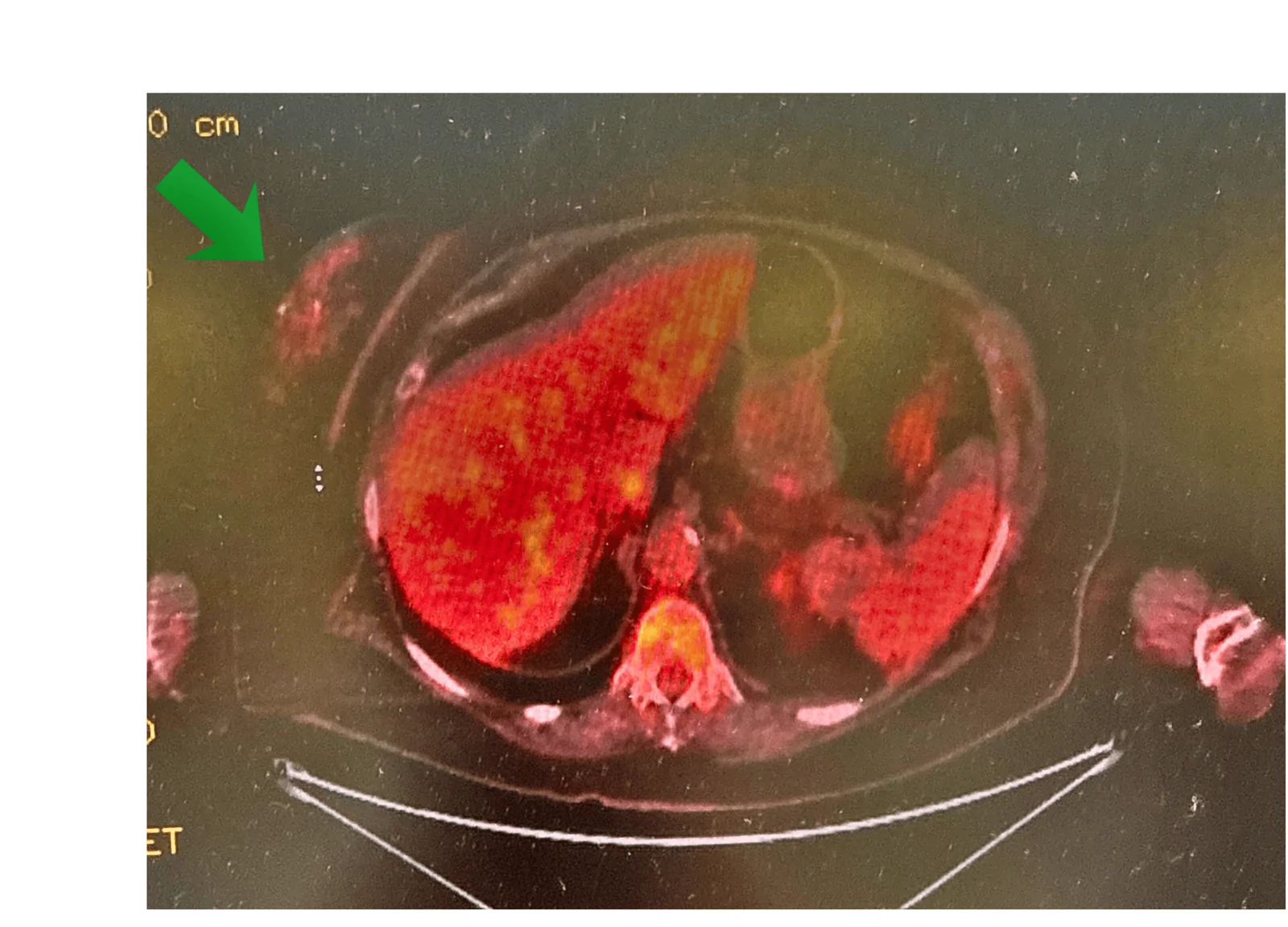
18F-FDG PET/CT demonstrating right breast and axillary disease Axial fused PET/CT image demonstrates FDG-avid right-sided breast and regional axillary disease, corresponding to the subsequently biopsied high-grade neuroendocrine carcinoma. 18F-FDG: fluorine-18 fluorodeoxyglucose, PET/CT: positron emission tomography/computed tomography

PET-CT in the fall of year 7 showed FDG-avid right breast and right axillary adenopathy (Figures 1-2). A repeat right diagnostic mammogram and ultrasound showed a 3.8 × 5.3 × 2.8 cm segmental area of a lobulated cluster of various-sized oval masses in the right breast, upper outer quadrant, 8 o'clock to 9 o'clock, anterior, at middle to deep depth, along with progression of right axillary lymphadenopathy. Visualized enlarged right axillary lymph nodes measured 1.8 cm, 2.3 cm, and 1.8 cm. The first two nodes noted were previously measured at 1.2 cm and 1.5 cm in the summer of year 7. Results were highly suggestive of malignancy, and a biopsy of the new breast lesion was recommended.

An ultrasound-guided biopsy of the right breast was completed in the fall of year 7, with pathology reporting that the histologic and immunophenotypic findings were compatible with a primary small-cell neuroendocrine carcinoma of the breast with focal squamous differentiation. The predominant neuroendocrine component showed small-cell features. The tumor was divided into two parts, A and B. Both parts demonstrated similar findings in the breast, with focal squamous differentiation and invasive carcinoma measuring 11 mm in greatest contiguous extent. Immunohistochemical studies performed on Part B demonstrated that the tumor had membranous reactivity with E-cadherin and p120 catenin. The tumor was diffusely positive for INSM1, synaptophysin, and p63. There was patchy reactivity for CK7, patchy reactivity to CK5/6, and rare reactivity to PAX8. The tumor was negative for CDX-2, TTF-1, SOX10, and GATA-3. A smooth muscle myosin heavy chain immunostain showed no definitive evidence of an in situ component. The tumor was estrogen receptor-negative (<1%), HER2-negative (IHC score 0), and had a high Ki-67 proliferative index (91-100%). With appropriate clinical and imaging correlation, the findings suggested a breast primary. Table 1 summarizes the timeline of the left and right breast lesions.

**Table 1:** Brief timeline review of left and right breast lesions CDX-2: caudal type homeobox 2, CK5/6: cytokeratin 5/6, ER: estrogen receptor, GATA-3: GATA binding protein 3, HER2: human epidermal growth factor receptor 2, IHC: immunohistochemistry, IDC: invasive ductal carcinoma, INSM1: insulinoma-associated 1, Ki-67: Ki-67 proliferation marker, p63: tumor protein p63, PR: progesterone receptor, TTF-1: thyroid transcription factor 1

Feature	Prior left breast tumor	Right axillary lesion	Right breast lesion
Date	Year 1	Year 7, summer	Year 7, fall
Histology	Infiltrating ductal carcinoma	High-grade neuroendocrine carcinoma, favor small-cell	Primary small-cell neuroendocrine carcinoma of the breast with focal squamous differentiation
ER	100%	0%	<1%
PR	30%	0%	Negative
HER2	Negative	0	0
Ki-67	10%	80%	91-100%
Morphology	IDC	Small-cell features	Small-cell neuroendocrine + focal squamous
Key IHC	-	INSM1+, synaptophysin+, TTF-1−, GATA-3−, CDX-2−	INSM1+, synaptophysin+, p63+, CK5/6 patchy, TTF-1−, GATA-3−, CDX-2−

Based on pathological findings, particularly the predominant neuroendocrine component, the patient was planned to start on a combination chemotherapy regimen of cisplatin and etoposide for 10-12 cycles. Surgical intervention was discussed with the patient. However, given her history and the presentation of a new, distinct contralateral breast tumor, systemic therapy was selected as the primary treatment approach. The patient also scheduled a consultation with a radiation oncology specialist for possible later interventions if warranted. The patient reported no significant concerns or complaints before the start of chemotherapy.

## Discussion

A primary small-cell neuroendocrine carcinoma of the breast with focal squamous differentiation is a rare type of breast cancer, and there is limited research and few large-scale studies available. A review of the available literature identified similar cases primarily in case reports and small case series [14-20]. When discussing neuroendocrine tumors, the lungs or gastrointestinal tract are typically the sites of origin, making the breast an unusual primary site [21]. Neuroendocrine markers are not used in standard protocols for breast cancer diagnosis, with differences in reported prevalence rates typically attributed to a lack of uniform histological and immunohistochemical (IHC) diagnostic criteria [22]. When diagnosing a predominantly neuroendocrine carcinoma of the breast, it is necessary to consider the possibility of the lesion(s) being a secondary tumor within the breast [16]. Site-specific IHC markers can help to distinguish between neuroendocrine carcinoma of the breast and metastatic neuroendocrine carcinoma from the lungs or gastrointestinal primary sites. TTF-1 is usually positive in ~70% of lung metastases, while CDX-2 is positive in 80%-100% of gastrointestinal metastases, and both are usually negative in breast neuroendocrine carcinoma [22]. GATA-3 can be positive for primary neuroendocrine carcinoma of the breast and negative for secondary tumors; however, it must be emphasized that GATA-3 is non-specific for neuroendocrine carcinoma of the breast and has been reported in other cancers, such as urothelial carcinomas, mesotheliomas, squamous cell carcinomas, and renal epithelial tumors [23]. With molecular diagnostics and characteristics, neuroendocrine carcinoma of breast tumors is typically hormone receptor-positive (estrogen receptor/progesterone receptor positive) and HER2-negative; however, there are limited data regarding the mutational profile [22]. As demonstrated in this case, the patient’s tumor was negative for TTF-1, GATA-3, estrogen receptor, progesterone receptor, and HER2, which may be related to the tumor's squamous component or other molecular alterations [24]. Taken together with the dominant right breast lesion, ipsilateral axillary involvement, absence of an identified extramammary primary on available imaging, and the overall clinicopathologic presentation, a primary breast origin was favored, although metastatic neuroendocrine carcinoma cannot be excluded solely based on immunohistochemistry.

Positive staining for neuroendocrine markers supports neuroendocrine differentiation. In this case, the tumor was diffusely reactive for synaptophysin, INSM1, and p63, while showing patchy or weaker reactivity for CK7, CK5/6, cytokeratin CAM5.2, and PAX8. Synaptophysin is a protein frequently used in immunohistochemical staining to confirm the neuroendocrine nature of a tumor [25]. INSM1 is a protein that acts as a transcriptional repressor involved in the development of neuroendocrine cells, which is highly expressed in neuroendocrine cancers and not typically in normal adult tissues or other tumor types [26]. p63 is expressed in the nuclei of myoepithelial cells of normal breast ducts and lobules (intraductal component), and its expression can be used to differentiate various types of neuroendocrine carcinomas and the primary site of origin [27]. CK7 positivity combined with p63 supports the conclusion of a primary breast tumor, as many metastatic neuroendocrine tumors involving the breast, such as those of pulmonary origin, lack this immunophenotypic pattern [15]. CK5/6 is a protein marker used to identify squamous cell carcinoma, as it is expressed in >95% of cases and helps differentiate basal-like breast cancer from other triple-negative breast cancers (TNBCs) [28]. Cytokeratin CAM5.2 is a monoclonal antibody that identifies cells of epithelial origin, particularly secretory or glandular epithelium, and is typically positive when combined with CK7 [29]. PAX8 is a marker commonly used in the diagnosis of carcinomas arising from the ovaries, kidneys, and thyroid gland but has recently been determined to be positive in 6%-41% of intermediate- or high-grade breast cancers, frequently those with a triple-negative phenotype, which is possibly related to the aberrant expression of PAX8 [30].

Upon reviewing the pathology results and staining patterns of this case, a predominantly neuroendocrine component and focal squamous differentiation were identified. A mixed primary breast tumor with these two distinct areas of differentiation is extremely uncommon, with only four similar cases found in a review of the literature [31-34]. An additional unusual feature of this case is the development of the contralateral nature of the new primary small-cell neuroendocrine carcinoma of the breast with focal squamous differentiation years after being diagnosed with and treated for IDC in the opposite breast. A review of the literature identified only one other similar reported case, which describes a 58-year-old woman who developed a primary small-cell neuroendocrine carcinoma in one breast 2.5 years after being treated for IDC in the contralateral breast [35].

Because this tumor type is rare and comparable clinical scenarios are limited in number, outcomes and treatment protocols are not standardized. When considering the management of a complex case such as this, guidance from established breast pathology is crucial. It has been determined that when neuroendocrine carcinoma of the breast coexists with adenocarcinoma or squamous cell carcinoma, the clinical behavior of these cases is determined by the neuroendocrine component due to the aggressive clinical behavior and poorer prognosis attributable to the neuroendocrine component [36,37]. Consequently, the regimen administered is similar to NEC alone. Surgical resection should be noted as the recommended first-line treatment for all breast neuroendocrine tumors; however, clinical history and classification as a contralateral breast tumor make this directive a less definitive option [38]. Chemotherapy should include the standard regimen used in the treatment of breast cancer, such as FAC (fluorouracil, Adriamycin (doxorubicin), and cyclophosphamide), CMF (cyclophosphamide, methotrexate, and fluorouracil), or a platinum-based agent plus etoposide, since the tumor biologic markers are similar to those of small-cell lung cancer. Hormonal therapy may also be added if the tumor expresses receptors [39,40]. The long-term survival benefit of etoposide plus cisplatin or carboplatin (EP) adjuvant chemotherapy for neuroendocrine carcinoma has been extrapolated from small-cell lung cancer treatment, with the number of chemotherapy cycles based on patient tolerability and the clinical scenario. The survival benefit of adding concurrent or sequential radiation to EP chemotherapy is also extrapolated from limited-stage small-cell lung cancer treatment data. Therefore, chemoradiation could be considered in selected patients with locally advanced disease (i.e., T3-T4 or lymph node involvement) [22].

Given the predominant small-cell neuroendocrine morphology, the patient was planned for platinum-based chemotherapy with cisplatin and etoposide. The planned regimen was extrapolated from treatment approaches used for small-cell neuroendocrine carcinoma; however, the optimal regimen and duration for primary breast neuroendocrine carcinoma are not established. Treatment decisions were made in the context of the patient's medical history, the tumor's nature, and the patient's overall goals. Concomitant chemoradiotherapy to the breast was not recommended to avoid increased toxicity. Given the tumor's rarity and its status as a contralateral primary, no definitive treatment protocol exists. Additional case reporting may help define appropriate therapeutic protocols.

## Conclusions

This case describes a rare contralateral primary small-cell neuroendocrine carcinoma of the breast with focal squamous differentiation and associated right axillary lymph node involvement, occurring approximately seven years after treatment of a biologically distinct left breast carcinoma. The tumor demonstrated small-cell morphology, diffuse neuroendocrine marker expression, focal squamous differentiation, and a high proliferative index. Clinicoradiologic and pathologic findings favored a new primary breast carcinoma; however, the absence of a definitive in situ component and molecular clonality analysis prevents the definitive establishment of breast origin. Given the rarity of this mixed phenotype, diagnostic distinction from metastatic neuroendocrine carcinoma and other high-grade breast carcinomas remains challenging. This case highlights the diagnostic and therapeutic uncertainty associated with this uncommon presentation. However, it does not provide evidence regarding treatment efficacy, tolerability, or a preferred treatment regimen; additional case reporting may help address these knowledge gaps.
